# Expression Cloning of Camelid Nanobodies Specific for *Xenopus* Embryonic Antigens

**DOI:** 10.1371/journal.pone.0107521

**Published:** 2014-10-06

**Authors:** Keiji Itoh, Sergei Y. Sokol

**Affiliations:** Department of Developmental and Regenerative Biology, Icahn School of Medicine at Mount Sinai, New York, New York, United States of America; University of Colorado, Boulder, United States of America

## Abstract

Developmental biology relies heavily on the use of conventional antibodies, but their production and maintenance involves significant effort. Here we use an expression cloning approach to identify variable regions of llama single domain antibodies (known as nanobodies), which recognize specific embryonic antigens. A nanobody cDNA library was prepared from lymphocytes of a llama immunized with *Xenopus* embryo lysates. Pools of bacterially expressed cDNAs were sib-selected for the ability to produce specific staining patterns in gastrula embryos. Three different nanobodies were isolated: NbP1 and NbP3 stained yolk granules, while the reactivity of NbP7 was predominantly restricted to the cytoplasm and the cortex. The isolated nanobodies recognized specific protein bands in immunoblot analysis. A reverse proteomic approach identified NbP1 target antigen as EP45/Seryp, a serine protease inhibitor. Given the unique stability of nanobodies and the ease of their expression in diverse systems, we propose that nanobody cDNA libraries represent a promising resource for molecular markers for developmental biology.

## Introduction

Since their discovery over a hundred years ago, antibodies have been broadly used in cellular and developmental biology, serving as unique tools for studying protein expression, localization and function. Antibodies are useful molecular markers for different embryonic tissues, for many types of cells composing these tissues, and for diverse subcellular compartments and organelles. Furthermore, specific antibodies are critical for structural and functional studies [1], [2], [3], [4], [5].

Due to the significant effort involved in the production and maintenance of conventional antibodies, generation of recombinant antibodies presents a useful alternative approach. However, since the antigen recognition site of conventional immunoglobulins is assembled from independently encoded heavy and light chains, the utility of a single recombinant immunoglobulin chain or even a fusion of heavy and light chain variable regions is tempered by poor stability and modest affinities of these antigen-binding derivatives [4], [6], [7], [8]. By contrast, the antigen-recognition site of naturally occurring single domain antibodies from llamas and camels is composed of a single variable region (nanobody), which is exceptionally stable and has an affinity comparable to that of conventional antibodies [9], [10], [11], [12]. Nanobody cDNA libraries can be easily expressed and maintained in bacterial and eukaryotic systems [10], [11] and the small size of nanobodies makes them a convenient tool for functional interference studies *in vivo* [13], [14], [15].

Due to their unique properties, nanobodies have a potential to replace conventional antibodies in structural biology, gene regulatory network analysis and signal transduction studies in the future [12], [16], [17], [18]. Given the shortage of antibodies that recognize *Xenopus* proteins, we decided to evaluate the potential use of nanobodies as molecular markers for embryonic cells and tissues. Here, we describe an expression cloning screen that resulted in the isolation of several nanobodies, which specifically recognize *Xenopus* embryonic antigens. Based on our proof-of-principle approach, we conclude that large-scale nanobody libraries will be useful for future structural and functional studies of the embryo proteome.

## Results

### Screening of nanobody pools by immunostaining of embryonic tissues

**Figure 1:**
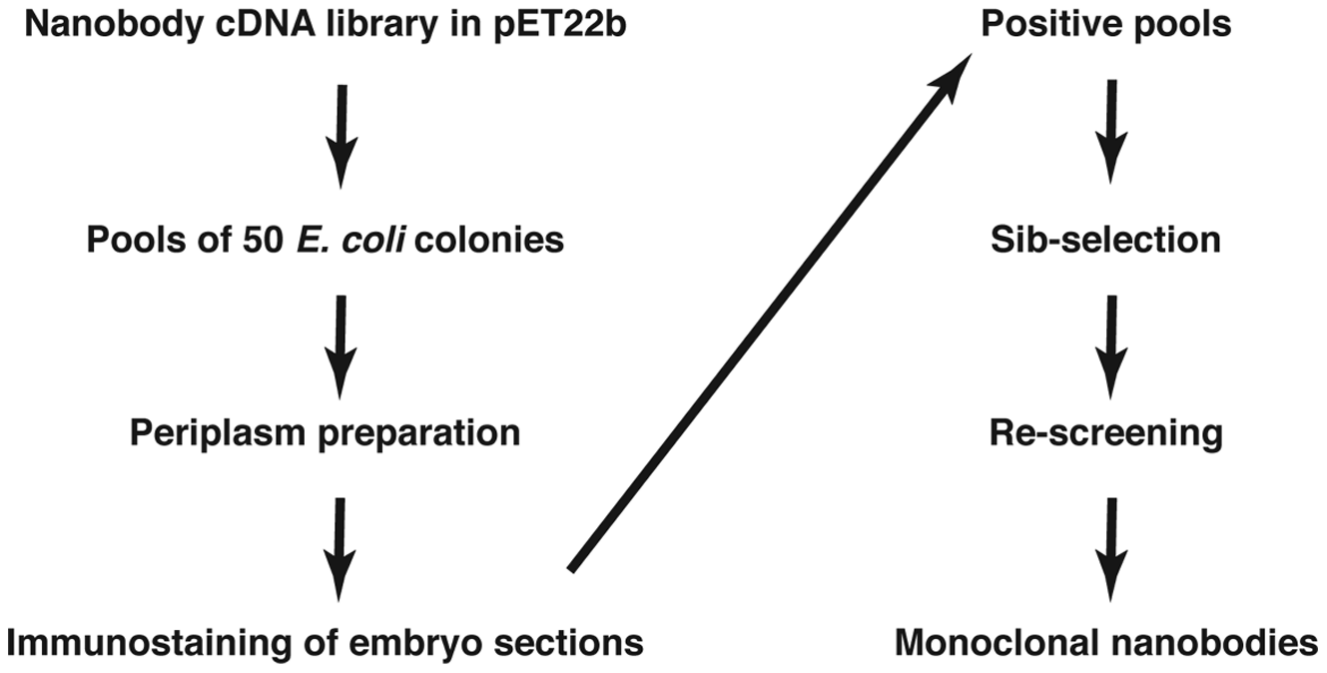
Expression cloning of nanobodies specific for *Xenopus* embryonic antigens. A scheme of the approach is shown. A nanobody cDNA library is made for bacterial expression using nested PCR from lymphocytes that were isolated from a llama immunized with *Xenopus* embryo lysates. Periplasm extracts prepared from pools of 50 bacterial colonies are used to stain embryo cryosections. Sib-selection of the positive pools leads to the identification of specific nanobodies.

To generate nanobodies specific to *Xenopus* embryonic antigens, we chose to use an expression cloning approach, in which pooled nanobodies are screened for their ability to stain cryosections of *Xenopus* gastrulae (Figure 1). The cloned nanobodies containing the pET22 vector-derived carboxy-terminal hexa-histidine tag can be detected with a specific antibody. Since immunization results in the selective proliferation of antibody-producing cells, we thought that testing a relatively few cDNA clones should be sufficient to identify specific nanobodies against *Xenopus* embryonic antigens. We chose to analyze pools of 50 colonies, arguing that the antibody titer in our crude periplasm preparations is likely to exceed 1∶50–1∶100. Sib-selection of positive pools and repeated screening allows the isolation of individual monoclonal nanobodies (Figure 1).

**Figure 2:**
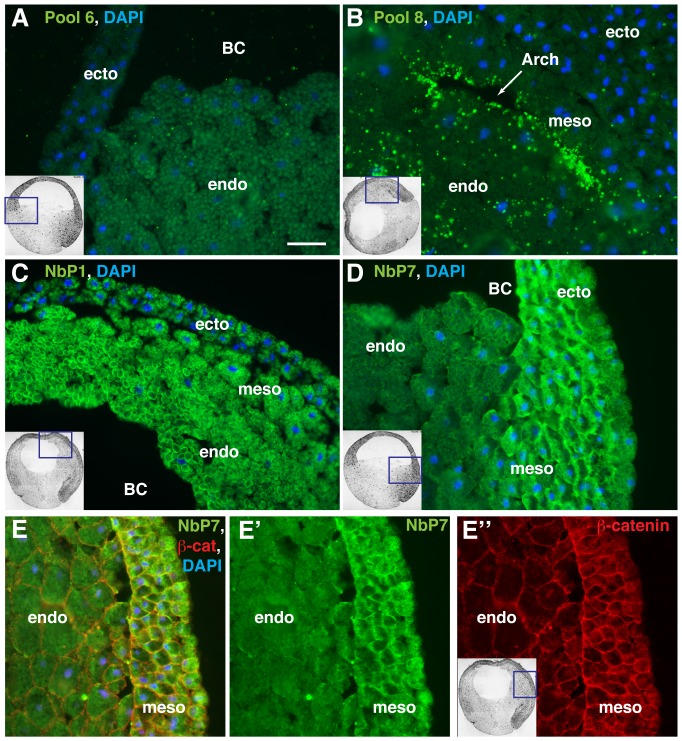
Immunostaining of gastrula embryos with pooled and individual nanobodies. Cryosections of *Xenopus* gastrulae were immunostained with periplasm (A, B) or individual nanobodies (C–E). A, Periplasm from pool 6 is a negative control. B, Periplasm from pool 8 shows punctate staining adjacent to the archenteron (Arch, arrow). C, NbP1 stains yolk platelets. D, NbP7 stains the cytoplasm and the cortex of both ectoderm and mesoderm cells. E. Gastrula section was costained with NbP7 (E′) and β-catenin (E″). Schematic insets indicate the approximate embryo region shown. Ecto, ectoderm, Meso, mesoderm, Endo, endoderm, BC, blastocoel. Scale bar is 50 µm.

Out of the 16 pools screened, we selected four positive pools, which specifically stained embryonic tissues. The majority of pools did not reveal any specific staining patterns and served as negative controls, e. g. pool 6 (Figure 2A). Pool 8 stained large non-specific aggregates on embryonic sections and was not studied further (Figure 2B). Pools 1 and 3 revealed predominantly endodermal staining of yolk granules, whereas pool 7 stained the cytoplasm and the cortex of the ectoderm. Sib-selection of the positive pools using smaller number of colonies (8–10) resulted in the isolation of nanobodies with similar staining patterns (Figure 2C–E). The isolated nanobodies were named NbP1, NbP3 and NbP7 to reflect their origin from periplasm pools 1, 3 and 7. Although the total number of the currently identified nanobodies is limited, these results show that our expression cloning approach can successfully generate cell and tissue-specific molecular markers that are suitable for immunostaining.

### Purification and characterization of the isolated nanobodies

**Figure 3:**
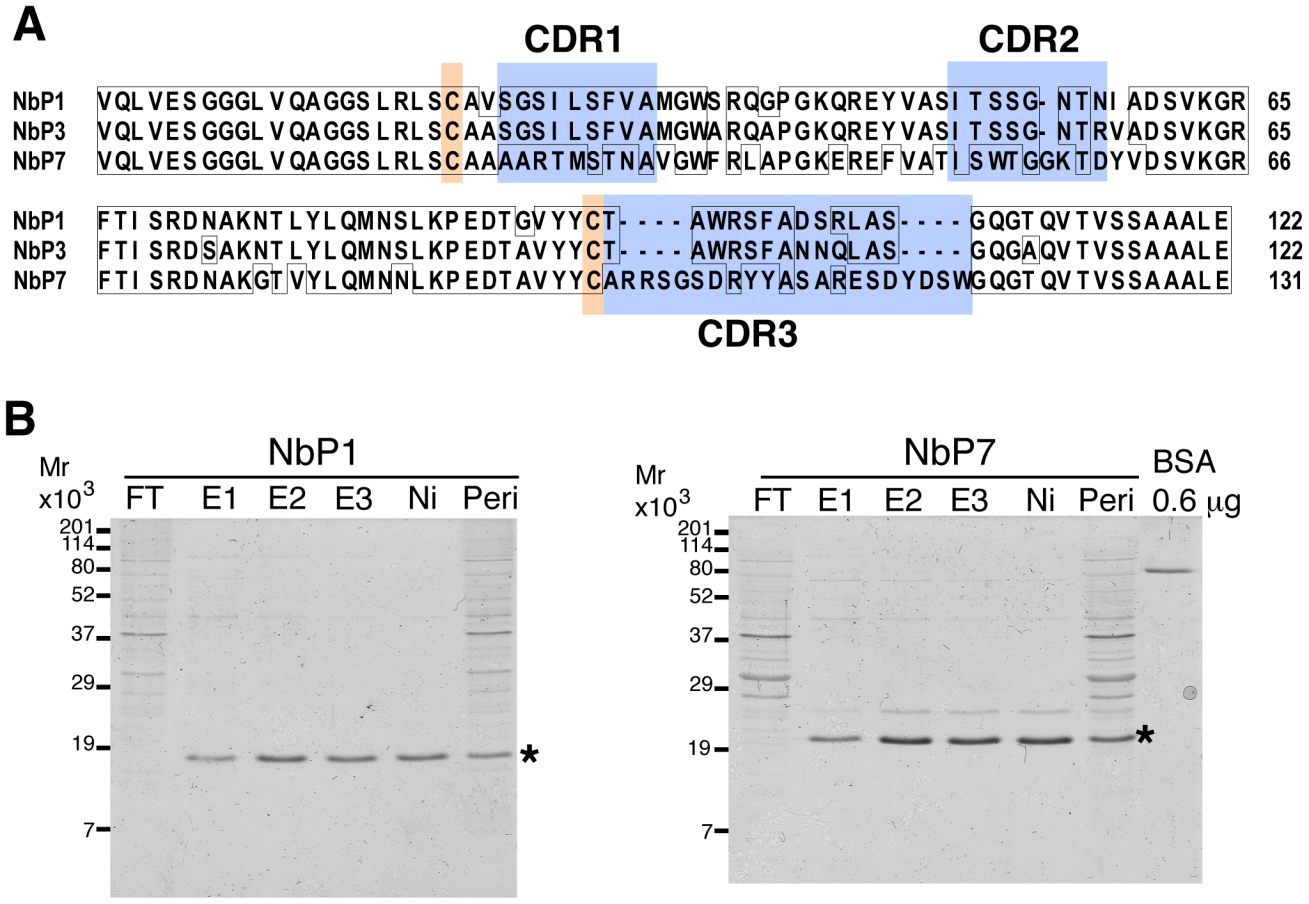
Monoclonal nanobody sequences and purification. A, Amino acid sequences of three isolated nanobodies are aligned. Two conserved cysteine residues and three complementarity-determining regions (CDRs) are indicated. B, Coomassie-stained gel demonstrating nanobody purification by Ni-chromatography. FT, flow through; E1–3, first to third elution; Ni, the Ni-resin before elution; peri, periplasm extract used for purification; BSA, bovine serum albumin. Asterisks indicate the positions of the purified nanobodies, NbP1 and NbP7.

The cDNAs encoding individual nanobodies were sequenced (Figure 3A) to reveal highly variable complementarity-determining regions (CDRs) and conserved cysteine residues, which are characteristic features of this class of antibodies [12], The two cDNAs corresponding to yolk-staining nanobody pools were very similar in their primary sequences; notably, the observed amino acid changes involved both the inter-CDR regions in addition to the CDRs. Monoclonal nanobodies encoded by the isolated cDNAs were purified to homogeneity by immobilized Ni-ion affinity chromatography [19] (Figure 3B).

**Figure 4:**
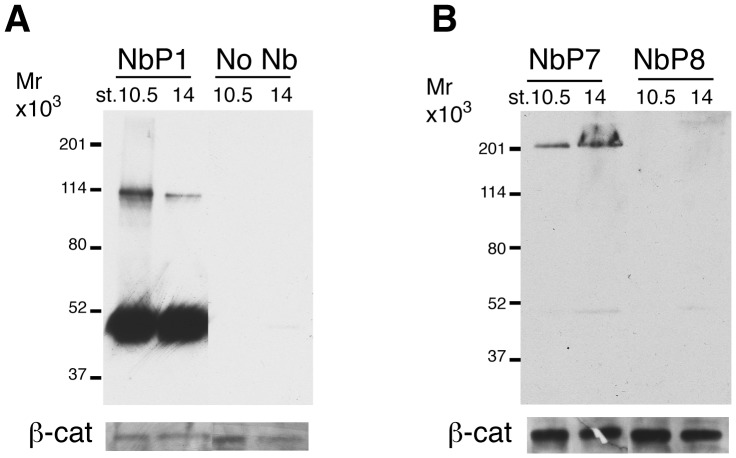
Immunodetection of specific antigens in embryo lysates. For western blot analysis, lysates of midgastrula (st. 10.5) and early neurula (st. 14) embryos were separated on SDS-PAGE gel, transferred to the Immobilon membrane and probed with different purified nanobodies, followed by anti-His tag antibodies. (A) NbP1 detects a major 47–49 kD protein and a minor 110 kD protein. (B) NbP7, but not NbP8, detects a 210 kD protein. β-catenin levels indicate protein loading.

Next, we assessed whether the purified nanobodies recognize their respective antigens by western blotting. The probing of *Xenopus* gastrula lysates with nanobodies, followed by incubations with anti-His-tag antibody and HRP-conjugated anti-mouse-IgG secondary antibody, revealed specific bands of approximately 47–49 kD for NbP1, and 200 Kd for NbP7 (Figure 4A, B). This analysis established the utility of the isolated nanobodies in immunoblotting, in addition to immunohistochemical staining.

### Identification of estrogen-regulated protein 45 as a molecular target of NbP1

**Figure 5:**
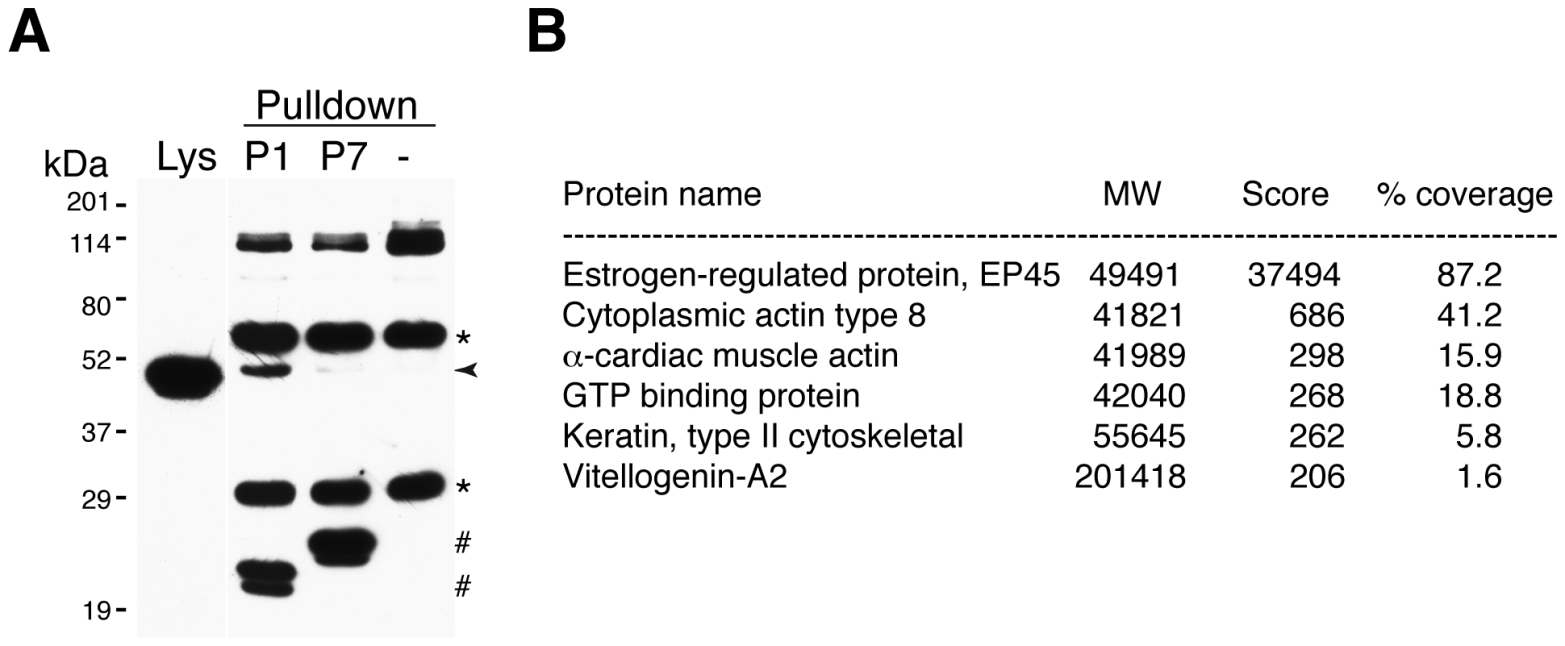
NbP1 immunoprecipitates a 49 kD protein from *Xenopus* embryos. A. NbP1 (P1) pull downs a specific protein from gastrula lysates (collected at st. 10.5–12). A normal embryo lysate (Lys) is shown on the left. NbP7 (P7) and no nanobody (-) groups represent negative controls. Immunoblot (probed with NbP1 and anti-His-tag) reveals heavy and light chains of the anti-His-tag antibody (asterisks), the nanobodies NbP1 and NbP7 (#) and the specific 47–49 kD protein band (arrowhead). B. Top candidate proteins detected by the LS-MS/MS analysis in a gel slice after immunoprecipitation by NbP1 from embryo lysates. Total scores based on the frequency of peptide representation, % coverage and molecular weights are indicated.

The reverse proteomic approach was next used to identify the antigen recognized by the isolated nanobodies. Embryo lysates were incubated with specific nanobodies and immune complexes were pulled down by an anti-histidine tag antibody bound by protein G-conjugated magnetic beads. Whereas NbP7-dependent pull downs failed to identify a specific antigen, NbP1 consistently precipitated a 49 kD protein (Figure 5A).

**Figure 6:**
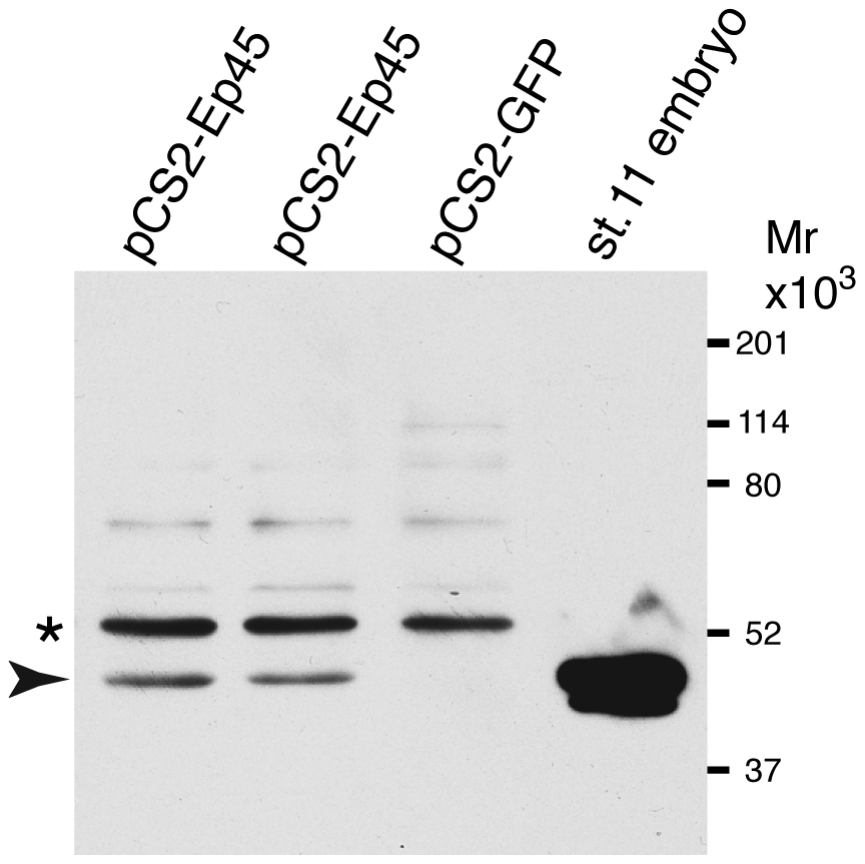
Estrogen-regulated protein EP45/Seryp is a target of NbP1. Semiconfluent HEK293T cells were transfected with the indicated DNAs. The cells were lysed 24 hrs later, and the protein lysates were separated on SDS-PAGE for imunoblot analysis with NbP1, followed by an anti-His tag antibody. NbP1 detects EP45 (arrowhead) in lysates of cells expressing pCS2-Ep45, but not pCS2-GFP. Asterisk points to a nonspecific band, which reflects loading. Embryo (stage 11) lysate is on the right, with two visible bands, as a positive control.

The gel slice containing the 49 kD protein band was subjected to the LC-MS/MS analysis (Keck laboratory, Yale University). The top hit of this analysis was the estrogen-regulated protein EP45/Seryp, a *Xenopus* yolk protein, which is the likely antigen recognized by NbP1 (Figure 5B). To further examine this possibility, HEK293T cells, which do not express EP45, were transfected with a plasmid DNA encoding EP45 or GFP as a negative control. Western blot analysis revealed a 49 kD band in the lysates derived from the EP45-expressing cells (Figure 6). Two protein bands of similar mobility were visualized in embryonic lysates, in agreement with proteolytic processing of EP45 [20], [21]. We conclude that EP45 is the antigen recognized by NbP1.

## Discussion

Our small-scale expression cloning approach based on immunostaining of embryonic tissues has led to the isolation of two specific nanobodies that recognize yolk platelets and one with the reactivity towards the cell cytoplasm and cortex. These nanobodies are easily produced in milligram quantities in *E. coli*, and can stored for prolonged periods until needed [12], [22]. These features make nanobodies ideal tools for the systems-level analysis of the existing proteomes [4], [6]. Similar expression cloning screens can be devised, based on staining of diverse embryonic tissues, specific developmental stages or based on the functional interference with a certain developmental process. Such screens together with more traditional phage display library screens [23], [24] will help create a large bank of nanobodies, with many applications in developmental and cell biology research.

Our study demonstrates that NbP1 functions in immunoprecipitation, western blotting and immunohistochemistry, contrary to the opinion that nanobodies are of limited use for the detection of denatured proteins [18]. The antigen recognized by NbP1 was identified as EP45/Seryp. Being one of the major yolk proteins, EP45 presumably functions as a serine protease inhibitor during oocyte maturation [20], [21], [25]. Yolk proteins are the main source of nutrition for *Xenopus* embryos before tadpoles start feeding, but the regulation of yolk proteins during early development has been poorly understood [21]. Future analysis of general yolk metabolism and the specific roles of EP45 are likely to benefit from the use of NbP1.

Naturally occurring single chain antibodies have been discovered more than 20 years ago and have been used as diagnostic tools in a number of studies [12], [22]. Although nanobodies have been generated against many diverse individual antigens, e. g., EGF receptor, botulinum neurotoxin, HIV, G protein coupled receptors, and caffeine [26], [27], [28], [29], [30], the analysis of nanobody repertoire produced in response to complex antigens has been limited. We propose that immunization of camelids with composite protein mixtures will be useful for the isolation of a variety of molecular tools and cellular markers for future systems-level proteome analysis. Consistent with this view, multiple nanobodies have been recently isolated from a llama immunized with a mixture of plant seed antigens [31]. However, crude embryonic preparations are likely to produce limited immune responses, which are dominated by a small number of strong immunogens. To overcome this difficulty, a variety of fractionation techniques can be used to enrich the cytoskeletal, nuclear or cell surface proteins. This can be done at the early stage, during immunogen preparation, or later, during nanobody selection from the library. For example, subtraction of phage display libraries should select for the reactivity against stage-specific and tissue-specific antigens. The latter strategy will produce nanobodies to developmentally relevant proteins, such as dorsal and ventral germ-layer-specific markers or specific to a particular organ, such as liver, pancreas or heart. This strategy is likely to be successful as high-affinity nanobodies can be isolated even from naïve (non-immunized) animals [10], [12] [32]. Nevertheless, for weak immunogens, a traditional approach through immunization with purified proteins is warranted. The detailed analysis of llama immune repertoire based on single chain antibody diversity remains the subject of ongoing studies.

## Materials and Methods

### Ethics statement

This study was carried out in strict accordance with the recommendations in the Guide for the Care and Use of Laboratory Animals of the National Institutes of Health. The protocol 04-1295 was approved by the IACUC of the Icahn School of Medicine at Mount Sinai.

### *Xenopus* embryos, immunogen preparation

Eggs were obtained from *Xenopus* females after injection of human chorionic gonadotropin (HCG). *In vitro* fertilization, embryo staging and culture in 0.1× Marc's modified Ringer's solution (MMR) were carried out as described [33], [34].

To prepare embryonic lysates for immunization, a mixture of gastrulae and neurulae embryos was homogenized and fractionated by centrifugation at 1800 g for 15 min. Several layers became visible after the fractionation, including yolk platelets, pigmented cytoplasm, clear cytoplasm and the lipid layer (from bottom to top) as described previously [35]. Clear cytoplasm was collected and used for llama immunizations (400 µg/injection) at Triple J Farms (WA).

### Nanobody cDNA library construction

Whole blood was obtained from the llama immunized with *Xenopus* embryo lysates. The blood was diluted with PBS (1∶1), layered on the top of Lympholyte (Cedarlane labs) and centrifuged for 20 min at 800 g for white blood cell isolation. Total RNA was prepared from 5×10^7^ cells using RNAeasy kit (Qiagen). Next, cDNA was made from 4 µg of total RNA with first strand cDNA synthesis kit (Invitrogen), following manufacturer instructions. DNA fragments corresponding to the variable heavy chain (VHH or nanobody), were amplified by Pfu polymerase using nested PCR primers specific to the conserved nanobody regions as described [30], [36] with modifications. First PCR reaction was carried out with the following two sets of primers: Ryc-Fw 1, 5′-GTCCTGGCTGCTCTTCTACAAGG-3′ and Ryc-Rv 1, 5′-GGTACGTGCTGTTGAACTGTTCC 3′; and Lad-Fw 1, 5′-GAK GTS CAG CTG CAG GCG TCT GGR GGA GG-3′ and Lad-Rv 1, 5′-CGC CAT CAA GGT ACC AGT TGA-3′. The PCR products (600–800 bp size) were subjected to the second PCR reaction with: Ryc-Fw 2, 5′-TA TAG AAT TCA GTG CAG CTG GTG GAG TCT G-3′ and Ryc-Rv 2, 5′-TTA TGC GGC CGC CGA GGA GAC GGT GAC CTG GGT-3′; Lad-Fw 2, 5′-A ATG AAT TCC GAK GTS CAG CTG CAG GCG-3′ and Lad-Rv-2, 5′-AT TGC GGC CGC TGA GGA GAC GGT GAC CTG-3′. The final PCR products sized between 360 to 430 bp were purified, double digested with Not1 and EcoR1 and ligated into the Not1- and EcoR1-digested pET22b vector (Novagen). This vector encodes for a carboxy-terminal His tag, which allows one-step protein purification by Ni-ion affinity chromatography. Additionally, His-tag has been used for nanobody detection, since nanobodies represent poor immunogens [12] and high affinity secondary antibodies to llama variable heavy chain fragments are not commercially available. After the ligated material was transformed into competent *E. coli* TG1 host cells, the presence of nanobody-specific inserts was confirmed by direct sequencing of plasmids purified from four random colonies.

### Nanobody purification

Pools of 50 TG1 colonies transformed with nanobody cDNA-containing plasmids were collected. Plasmid DNA was isolated from the pooled colonies and re-transformed into BL21Star cells. These were cultured in 5 ml of LB media containing 50 µg/ml Ampicillin until the cultures reached the OD of 0.4–0.6. Protein expression was induced by 0.5 mM of IPTG for 5–6 hrs at 37 degrees. The pellets were suspended in 100 µl of TES buffer (0.1 M Tris, pH 8.0, 1 mM EDTA, 0.5 M Sucrose, 1 mM PMSF, 0.02% NaN_3_) on ice for 30 min. For osmotic shock, 150 µl of cold 0.2× TES buffer were added to the suspension for another 30 min. Periplasm protein-containing supernatant was collected after centrifugation at 14000 rpm for 20 min, added with NaCl to 300 mM final and stored at 4 degrees. These periplasm preparations were used for immunostaining of sectioned embryos as described below.

Monoclonal nanobodies were purified by immobilized Ni-ion affinity chromatography using His60-Superflow resin (Clontech) according to the manufacturer's instructions. After elution with 250 mM imidazole (pH. 8.0), eluates were collected and dialyzed in 0.1 M NaHCO_3_ (pH. 8.3) and 0.5 M NaCl. The plasmids corresponding to specific nanobodies have been sequenced and are available through Addgene and upon request.

### Immunostaining of embryo cryosections

For cryosectioning, embryos were devitellinized at stages 10, 11 and 12.5, fixed in MEMFA for 1–2 hrs, and washed with PBS. Samples were re-fixed in Dent's fixative for 1–2 hrs, washed with PBS and embedded in 15% fish gelatin/15% sucrose solution as described [37]. The embedded embryos were frozen in dry ice and sectioned at 10 µm with Leica CM3050 cryostat. Cross-sections of stage 10–12.5 gastrulae were probed with periplasm preparations of nanobody pools (1∶2) or with purified nanobodies (1.5–5 µg/ml of NbP1; 15–60 ng/ml of NbP7), followed by the anti-His-tag mouse monoclonal antibody (Abcam, 1∶1600) and anti-mouse IgG conjugated with Alexa488 (Invitrogen, 1∶500). Co-staining of sections with polyclonal rabbit antibodies to β-catenin (1∶200, Santa Cruz) was developed by secondary anti-rabbit IgG conjugated with Cy3 (1∶200, Jackson ImmunoResearch). Images were captured on an AxioImager microscope, using the AxioVision software (Zeiss).

### Cell culture and transfection

HEK293T cells were cultured in Dulbecco's modified Eagle's medium (GIBCO) supplemented with 10% fetal bovine serum (Gemini-Bioscience) and were transfected with plasmids using linear polyethylenimine (MW 25,000; Polysciences) as described [38]. Briefly, 8 µl of polyethylenimine stock solution of 1 mg/ml in water (pH 7.0) were mixed with 1–2 µg of pCS2-EP45 (Marteil et al., 2010) or control pCS2-GFP DNA and the mixture was added to the culture medium of semiconfluent HEK293T cells in a six-well plate. After 24 hr culture, the transfected cells were lysed in each well with 200 µl of SDS sample buffer for protein analysis.

### Immunoblot analysis and immunoprecipitation

Western analysis was carried out using standard techniques as previously described [39]. Briefly, lysates containing the equivalent of 0.2–0.4 *Xenopus* embryos or 1/40 of 6-well-plate lysates of transfected HEK293T cells were separated on 8–10% SDS-PAGE, the proteins were transferred to the PVDF membrane and incubated overnight with a nanobody (0.2–0.35 µg/ml) at 4°C followed by incubations with anti-6His mouse monoclonal antibody (Genetex, 1∶2000) and the secondary anti-mouse antibody conjugated with horseradish peroxidase (Jackson Immunoresearch, 1∶2000). The signal was developed by enhanced chemiluminescence as described [38].

For pulldown experiments, nanobodies were incubated with lysates prepared from 300–400 gastrula embryos in 1% Triton-X100, 50 mM Tris, pH 7.5, 1 mM EDTA, 50 mM NaCl for overnight at 4 degrees. Incubation was followed by the addition of anti-His-tag antibodies (GeneTex) and the immune complexes were batch-purified on the Protein G coupled to magnetic beads (Life Technologies). After washing with lysis buffer, the beads were boiled 3 min in the SDS-sample loading buffer, and the proteins were separated using 8% SDS-PAGE [40], followed by SimplyBlue (Invitrogen) staining or by immunoblot analysis. Mass spectrometry analysis was carried out at the Keck MS lab (Yale University).
